# Report of the Fourth Meeting of the British Association for the Advancement of Science; Held at Edinburgh, in 1834

**Published:** 1836-04

**Authors:** 


### Art. II.

*Report of the Fourth Meeting of the British Association for the Advancement of Science; held at Edinburgh, in* 1834.—*London,* 1835. 8vo. pp. 700.

The establishment of the British Association is likely to form an important epoch in the history of science, and, with reference to that part of it in which we are more immediately interested, the most beneficial results may be anticipated. "Sine philosophiâ trunca et debilis foret medicina" was the saying of the great Roman physician; but, though its truth has seldom been called in question, it has been too generally lost sight of; and, even when kept in view, has not always been fortunately applied: witness the many instances in which other branches of philosophy have been made to supplant, rather than illustrate, the principles of medicine; in which physiology, the basis of all rational medicine, has been resolved into chemical, mechanical, or metaphysical hypotheses, to the exclusion of the actual observation of the laws of life. This has arisen from too partial a recognition of the relations of medicine to other departments of knowledge, each enquirer bringing it into apposition with that branch of science with which he happened to be most familiar, without considering that principles, in themselves true, are not equally applicable to every subject.

The study of every science has its own particular influence on the development of the mind, and generates peculiar aptitudes for the reception of truth, and peculiar liabilities to error: hence the sciences demand extensive aid of each other. It is not, indeed, either necessary or possible that the professor of one branch should be profoundly versed in all others, but he may nevertheless be sufficiently imbued with the spirit of universal science to exempt him from partial and exclusive habits of thinking. It is obvious that no science stands more in need of collateral aid than medicine, not only from its being one of the most arduous subjects to which the mind can be applied, but from the immense variety of knowledge which it concentrates; thus drawing from every source of observation, and exercising every species of reasoning: the adoption of medical philosophy into the great circle of the sciences, under the auspices of the British Association, is therefore an event to be viewed with much satisfaction by every enlightened cultivator of our art.

Among the papers contained in the volume before us there are two relating to medicine; one on the laws of contagion, the other on animal physiology.

I. *Report on the State of our Knowledge of the Laws of Contagion.* By William Henry, m.d. f.r.s. &c. late Physician to the Manchester Royal Infirmary and Fever Wards.

After some general remarks on the bearings of the subject, and a just animadversion on the controversial and disingenuous spirit by which too many writers have been actuated, Dr. Henry proceeds to illustrate the laws of contagion. The term contagion is used in its more comprehensive sense, without reference to the distinction attempted by some writers between *contagion* and *infection*. It is unnecessary for us to dwell on each of the laws here laid down, because many of them, though properly introduced into a connected survey of the subject, are too well established to admit of any discussion: several of them, however, refer to points of considerable moment which still remain undecided, and these we may notice without adhering precisely to the arrangement of our author.

*The Origin of Contagions.* It is well known that some contagious diseases, as typhus, frequently originate in the animal body, when subjected to the action of certain external causes, among which the most obvious are confinement in crowded and ill-ventilated places, deficient or unwholesome food, intemperance, excessive fatigue, long-continued exposure to cold and moisture, and depressing passions of the mind. Such diseases are also occasionally observed to arise sporadically, without the intervention of the causes above alluded to, and, to all appearance, independently of contagion. There is, however, a class of contagious diseases which have never yet been proved to arise sporadically, and are generally believed never to do so: the contagions which produce these are therefore called *specific contagions;* such are those of syphilis, measles, smallpox, cowpox, hooping-cough, scarlatina, &c.

Dr. Henry supports the common doctrine, that contagious diseases of a specific kind never originate spontaneously: this position he conceives to be strengthened by certain facts, which we will consider severally.

1. Specific diseases "have never been met with in any country, when visited for the first time, after having been previously shut out from intercourse with the civilized world."

The argument derived from this fact is somewhat invalidated by the consideration that a rude and scattered people, among whom the intercourse of life is comparatively rare, and whose habits approximate in some degree to those of nature, are less subjected to the causes from which contagious diseases of every kind are most likely to arise, than a people further advanced in the social progress: they are not exposed to the vitiated atmosphere of crowded cities, to the union of intemperance and want which destroys the health of a poor and immoral population, nor to the various anxieties and causes of mental depression with which the advantages of civilization are alloyed.

2. "The historical eras may be fixed when many of these first invaded the countries where they now prevail, and the line of their march may be distinctly traced out."

This is a powerful argument in favour of the propagation of such diseases by contagion, but it is no argument against their occasional origin from other sources.

3. "Specific diseases have been known to become extinct for a time in certain situations, and their revival has been traced unequivocally to a foreign source. Thus, the smallpox disappeared several times from the island of Minorca, apparently from having already attacked all who were liable to it. In one instance the interval extended to seventeen years; in another, after having been absent for three years, its return was clearly traced to the crew of a ship of war which had arrived from the Levant. Seven similar intermissions of the same malady are recorded to have happened at Boston, in New England; in three only of which the channels of its reintroduction could be discovered. But these three instances render it much more probable that the poison causing the disease should, in the remaining four, have been imported anew, than that it again should have been generated. For, though it cannot be denied that a poison may be again elaborated by a concurrence of the same circumstances which originally produced it, yet, in assigning causes, we must be guided by actual observations, and not by possible contingencies."

Here we think our author's reasoning turns against himself. It is admitted, with reference to a specific contagious disease, the smallpox, that it *may* originate independently of contagion: nay, it is certain that, in one instance at least, it must have done so, or it never could have originated at all. Again, it is admitted that it has been known to arise without the possibility of its being actually traced to a foreign source: it is moreover previously allowed by Dr. Henry, that in this country, where the disease is constantly prevalent to a greater or less extent, individuals are often affected with it when no source of contagion can be discovered: "it frequently happens that the most searching and diligent enquiry fails to trace a specific disease to its source. We are told that not one in twenty cases admitted into the Smallpox Hospital in London could be referred to any immediate original. In a few instances specific diseases have appeared within boundaries which might have been supposed to have perfectly excluded them. In the Penitentiary at Millbank, a prisoner was seized with smallpox, notwithstanding his apparently perfect insulation." Now, although no positive conclusion can be drawn from these premises, we would submit that, adopting our author's principle, and being guided by *actual observation*, we are led to infer that, although the disease in question be generally propagated by contagion, it is by no means improbable that it does occasionally originate in the human body, independently of this cause: the entire possibility of the latter occurrence being conceded, every instance in which it *appears* to take place goes so far in favour of the supposition that it *actually does* take place; and, although due allowance must be made for the influence of contagion in many cases where it cannot be traced, still a large number of cases in which a specific disease arises to all appearance spontaneously affords at least a probability that it really does so arise in *some* of them.

The instance of smallpox, however, is perhaps more favourable to the opinion espoused by our author, than that of several other diseases. With reference to measles, and still more to scarlatina and hooping-cough, every experienced practitioner must have met with instances so apparently sporadic, that, if his mind had not been prepossessed with the idea of contagion, he would never once have thought of attributing them to this source; and he must also have seen a number of cases of each of these diseases occur in a neighbourhood so simultaneously as almost to exclude the supposition of contagion as a probable cause of their appearance.

But, of all known diseases, certain affections resembling the venereal afford most countenance to the opinion that specific contagions do occasionally arise spontaneously.

John Hunter admitted the existence of anomalous animal poisons generated independently of infection, but producing effects sometimes so nearly resembling syphilis as not to be easily distinguished from it, and, like syphilis, communicable from one individual to another. Several very striking cases are given in his work on the Venereal Disease; and he was of opinion "that new poisons are rising up every day, and those very similar to the venereal in many respects, though not in all." (P. 387, 4to. ed.) Mr. Abernethy went so far as to declare that he knew of no criterion by which the spurious disease could in all cases be distinguished from the genuine. "Mr. Hunter," he observes, "seemed to wish the prosecution of this subject, probably from the expectation that some characters appropriate to these diseases might be detected. I have not, however, been able to discover any: the fictitious disease in appearance so exactly resembles syphilis, that no observation, however acute, seems to be capable of deciding upon its nature." (*Surgical Observations*, 8vo. 1824, p. 134.) We believe that the greater number of surgeons of the present day will be found much more disposed to extend than to dispute the observations of Abernethy on this point. We will not here hazard a suggestion of the possibility that two diseases, which cannot by any means be distinguished, may, in effect, be one and the same disease; and hence that actual syphilis may, even now, occasionally originate spontaneously: it is sufficient for our purpose that specific animal poisons are found, in the present day, to originate in the bodies of certain individuals, and to be communicated from these to others. If such be the fact, and there is little doubt that it is so, there seems no improbability in the supposition that those specific contagions, which have long been abroad among the species, may, under certain circumstances, be generated anew in the system of an individual.

We might strengthen the analogy by the instance of *hydrophobia*, were it not that some writers have doubted (though, as we think, without sufficient reason,) whether this disease ever arises, even in the canine race, independently of infection. It may also be objected, with more validity, that its origin does not come fairly within the limits of human pathology, because there is no ground for believing that it ever originates in man.

After all that has been said, we beg not to be understood as unconditionally adopting either side of this very difficult question: our only object in entering upon it has been to oppose a hasty conclusion, which has a tendency to preclude enquiry. If it be admitted as in any degree probable that specific diseases sometimes arise spontaneously, observation will be kept alive to the causes which may operate in their production; but, if it be too readily taken for granted that they never do originate in this manner, we exclude ourselves from an investigation which, though it appear barren at present, may afford important results in some more advanced era of medical science.

*Conversion of Simple into Contagious Fever.* "Of the nature of those processes," says Dr. Henry, "by which a simple fever becomes contagious in its progress, we are totally ignorant. The opinion that a contagious poison is, in any case, generated by a change in the animal fluids analogous to fermentation or to putrefaction, (a change veiled by Sydenham under the phrase *commotio sanguinis*), is inconsistent with general reasoning as well as with observation. The tendency to putrescence in the solids or fluids of the animal body, at temperatures favourable to that process in dead matter, is counteracted by the undefinable principle of life, so long as that principle retains sufficient energy. During a contagious fever, none of those gases are necessarily evolved, which are the constant products of animal putrefaction. A person sick of typhous fever, enjoying all the advantages of cleanliness and fresh air, and emitting no sensible odour, may yet impart a fatal infection. An instance is on record, in which a person under such circumstances was accompanied, for about half a mile, in a coach, by four individuals, none of whom perceived the slightest odour, but all caught the infection, and died in consequence. It may be remarked also, that the odours, which arise from persons labouring under acute specific diseases, are not similar to those of common putrefying matter, but are distinct and peculiar. When we add to these arguments, that the perversion of a vital process, such as that of secretion, occasions, in at least one decided instance (*rabies canina*), the formation of a poison, by an organ which commonly secerns a bland and harmless fluid, the weight of evidence must be allowed greatly to preponderate in favour of the opinion, that all morbid animal poisons are the results of vital operations; and that chemical changes, if concerned at all, are under the control of the vital principle." (P. 72.)

An interesting enquiry here suggests itself, namely, what other diseases are there which, like common fever, may occasionally become contagious, although they are not so under ordinary circumstances?

Erysipelas has sometimes prevailed as a formidable epidemy, and, even when sporadic, frequently assumes a disposition to spread by contagion: nor is this confined to the idiopathic form of the disease; experience sufficiently attests that erysipelas supervening on wounds may also become contagious, especially in hospitals. Catarrh and pneumonia have frequently presented themselves in an epidemic, and probably a contagious form.

It is also worthy of remark, that a vast variety of diseases have at different times occurred as complications of contagious fever, and formed characteristic features of particular epidemies: so great indeed has been their number, that we may almost conclude, with Sennert, "nihil fere symptomatum et malorum est quod non cum peste et malignis ac pestilentibus febribus interdum conjungatur." (*De Feb.* lib. iv. c. xviii.) It would seem, therefore, that there is nothing in the nature of such affections incompatible with their communication by contagion; since, in the instances alluded to, they are so influenced by the causes of contagion as to become communicable along with the fever they accompany. These considerations point to the possibility that a contagious principle may, under certain circumstances, be superadded to many diseases, and may sometimes operate in determining their frequency when its presence is very little suspected. It must have occurred to every practitioner to see a disease not generally esteemed contagious—as a sore-throat or a diarrhœa,—run through a whole family in a manner not easily to be accounted for; and, although "nec deus intersit" be an excellent maxim, it here applies no more to contagion than to "a peculiar constitution of the atmosphere," the operation of unknown local causes;" and other explanations which certainly do not possess the advantage of superior intelligibility: while, therefore, a belief in contagion is not hastily to be adopted in such instances, there is no reason why it should be absolutely excluded from consideration.

*Mode in which Contagions are communicated.* "Among contagious poisons," observes our author, "there are some that exist in a visible and tangible state, generally in that of liquids; others are not at all perceptible by our senses, and are known to us only by their effects. The liquid poisons are efficient, only when applied beneath the cuticle, or to parts where the cuticle is very thin, or to the surfaces of mucous membranes; and if immediately and completely washed off, they inflict no injury. The action of some of those poisons, of syphilis for instance, does not necessarily extend beyond the part to which they are applied. Other poisons, when inserted or inoculated, act locally in the first instance, and afterwards give rise to general febrile excitement, which is necessary to the formation of fresh poison in the inoculated part, or in the system. After inoculation for smallpox, the constitutional disturbance is generally well marked; in cowpox, often so faintly as to be scarcely distinguishable; yet even in the latter, some degree of general fever seems to be essential to the perfect state of the pustule. It is only at this period of full development (called the time of *maturation*) that the fluid contents of the pustule, (which in the cowpox is limpid, in smallpox purulent,) can be depended upon for producing its appropriate effect. Before maturation, the fluid is inert; after that period, it is sometimes effete, and sometimes produces a modified disease. All attempts to excite smallpox or cowpox, by inoculating with the blood or with any other animal fluid, have been unsuccessful.

"XI. When the liquid animal poisons are kept in a moist state, at temperatures not exceeding those of a warm atmosphere, they undergo spontaneous changes which materially affect their specific properties. Variolous matter, thus negligently preserved, has been known to produce a train of symptoms resembling those of smallpox, but yet giving no security against the return of that disease. But the liquid poisons, dried at the lowest temperature adequate to that purpose, may be kept in close vessels unimpaired for an indefinite time, and regain their infectious properties when moistened with very little water. The mixture of them, however, with a large proportion of water, renders them inefficient. Dr. Darwin relates that, in some experiments by Mr. Power, smallpox matter was found to be infectious after diffusion through five times its quantity of water; but that its dilution might be carried so far as to render it inert. This is precisely analogous to what happens with common poisons, the most virulent of which is disarmed of its noxious power when sufficiently diluted.

"XII. Of the chemical constitution of the liquid contagious poisons we are entirely ignorant; nor is it probable that the knowledge, if we possessed it, would throw any light on their mode of action. We are well acquainted with the composition of many poisons (the prussic and arsenious acids, for example), without at all understanding in what way they act so powerfully upon the animal system.

"XIII. Beside the liquid poisons, requiring contact for their operation, there is another class which are independent of that mode of communication, and are transmitted to small distances through the atmosphere. Such are those of scarlatina, measles, hooping-cough, chicken-pox, &c. In a few instances diseases imparted by contact are also caught by emanations or effluvia. The smallpox, it is well known, may be propagated in both ways; and the plague, certainly infectious at small distances, has, of late years, been proved to be communicable by inoculation with the matter of the glandular abscesses. Dr. White, after two unsuccessful attempts to inoculate himself, caught the plague by the third, and died in three days; and Dr. Valli, in 1803, fell a victim to a similarly rash experiment." (P. 73.)

To reason correctly on the subject of inoculation, it is essential that the meaning of this term be exactly defined. In smallpox and cowpox, a virus is elaborated which contains, as it were, the essence of the disease, and which, being inserted beneath the cuticle of a healthy person, excites a local specific action that is afterwards communicated to the system. This is inoculation in the proper sense of the term; but it is conceivable in the instance of a highly contagious malady that the insertion of any fluid derived from the diseased body, or even the application of such fluid to the surface of the cuticle, might excite the disease in a healthy individual. This might also be called inoculation; but it is evidently not so in the same sense as in the former case: it is, in reality, an instance of simple contagion. Now with respect to plague, it is well known that even extraneous substances impregnated in the smallest degree with the fluids or exhalations of the diseased body will readily communicate the disorder: surely, then, there is no improbability in the supposition that a small quantity of purulent matter derived immediately from the body of a patient should impart the disease quite independently of its containing a specific virus elaborated from the general system like that of smallpox. Plague seems to have been much too hastily associated with the exanthemata, from which it differs in the following striking particulars: in all known exanthems the eruption has a uniform character, and is thrown upon the skin or some other extensive membranous surface, while in plague the character of the eruption is various, and its seat irregular; it consists of tumours in the glands, of carbuncles involving both the skin and the subjacent textures, of tubercles situated in the cellular membrane, and other affections of less constant occurrence. The carbuncles have as good a right to be considered a part of the supposed exanthematous eruption as the buboes; and, if so, the plague has a *double eruption*, therein differing from all other exanthematous diseases. Lastly, the pestilential buboes and carbuncles follow no regular course of maturation and decline; they frequently appear several times in succession; nor do they seem like the eruptions of the exanthemata to have any determinate relation to the symptoms or progress of the disorder.

*Identity of Smallpox and Cowpox.* When speaking of the recurrence of contagious diseases, Dr. Henry remarks, that "smallpox and cowpox act as safeguards against each other; or, when (failing this) the one occurs in a person who has passed through the other, the second in order of sequence, whether smallpox or cowpox, assumes a modified, and generally a much milder form. There can be little doubt, however, that those two diseases are essentially the same. We have no evidence that any one specific disease affords a security against any other which is distinguished from it by marked characters and a different succession of symptoms. Neither smallpox nor cowpox gives a durable protection against measles, hooping-cough, or scarlatina." We are somewhat surprised at the affirmation that there can be little doubt of the identity of smallpox and cowpox. We cannot perceive any valid reason for entertaining such a belief. The alleged results of Sonderland's experiments are indeed striking; but the repetition of these experiments in other quarters has afforded so little countenance to the conclusion he derived from them, that we may be allowed to doubt the accuracy of the statements, although without impugning the veracity of the narrator.

Among the theoretical objections to the identity of the two diseases, there is one which we do not recollect to have seen noticed, but which appears to us to have some force: it is this. On the supposition that smallpox and cowpox are essentially the same, the difference of the phenomena must be ascribed to a modification effected in the action of the same virus, by the respective peculiarities of constitution in the human subject and the cow: if, therefore, the virus could be obtained *as yet uninfluenced by the constitution of either of these animals*, it might reasonably be expected to produce cowpox in a cow and smallpox in a man. Now the disease called the grease in a third animal, the horse, affords the means of making the experiment: the grease is known to be capable of generating cowpox in the cow, and ought, by hypothesis, to generate smallpox in a man. Such, however, is not found to be the case: the grease produces nothing but *cowpox*, whether in man or in the cow. It is, however, useless to theorize on this subject: we are in want of further experiments; and it is by no means improbable that a very extensive series of inoculations with the virus of smallpox, cowpox, and the whole family of pustular and vesicular exanthems, in their more characteristic as well as in their modified forms, with a patient comparison of their effects on man and other animals, might lead to the correction of some erroneous views, and the establishment of some important facts, in pathology.

*Fomites.* The following is a judicious summary of our knowledge on this subject.

"It has been long known that dry porous bodies, when exposed to the atmosphere, increase in weight by absorbing aqueous vapour. In like manner, there can be no doubt that contagious vapours or emanations are absorbed by porous substances, and are again exhaled in an active state. Boyle remarked that 'amber, musk, and civet perfume some bodies, though not brought into contact with them, as the same determinate disease is communicable to sound persons, not only by the immediate contact of one who is infected, but without it.' Contagious emanations, thus imbibed by porous bodies, have received the name of *fomites*. They are capable of issuing forth with unabated, and, it is even asserted on good authority, augmented activity. It is probable, therefore, that they are emitted in a state of increased concentration, the porous body having imbibed those vapours, in preference to the elastic fluids which constitute the atmosphere. The propagation of contagious poisons, in the state of fomites, is illustrated by the following among numberless similar instances:—1. The contagion of the plague of 1665 was conveyed in a box of clothes from London to Eyam, a small village in Derbyshire, out of whose scanty population it carried off two hundred and fifty persons. 2. Smallpox infection has been transmitted from London to Liverpool, by means of new apparel made in a room where persons were sick of that malady. 3. Dr. Hildebrant introduced the poison of scarlatina into Podolia, a distance of several hundred miles, by a suit of clothes, which he had worn at Vienna while attending persons sick of that disease, and had laid by for several months. 4. Of the propagation of a fever of the typhoid character by fomites, Sir John Pringle has recorded a striking example. A number of old tents, which had been used as bedding by soldiers sick of low fever, were, on the disembarkation of the troops at Ghent, sent to be repaired. Twenty-three Flemish workmen were employed in the business, out of whom seventeen took the fever and died, though they had no personal communication with the troops.

"XXIII. It has not been ascertained how long fomites may retain their activity; but there is reason to believe that in articles closely packed they may remain unaltered for several years. Sennertus relates an instance in which, after a violent plague at the city of Breslaw, in 1542, the pestilential contagion imbibed by linen cloth which was kept fo ded up, issued forth fourteen years afterwards in another city, and gave rise to a plague, which caused great devastation. In Dr. Parr's *Medical Dictionary* (art. CONTAGION), a fact is stated, which, if well authenticated, would indicate a much longer period for the durability of the contagion of plague.

"XXIV. The subject of fomites is well worthy of further investigation. Hitherto we have acquired no information respecting the comparative powers of different porous bodies to absorb contagion. Technical distinctions into 'more or less susceptible articles' are, it is true, recognized by the quarantine laws; but they appear to be founded on loose analogies rather than on careful observations. 1. It is extremely probable that *different* porous bodies vary as to their powers of absorbing the same contagious emanation, as we know that they differ in their powers of imbibing a given elastic fluid. 2. In the same porous body, it is quite conceivable also, that the power of absorbing different contagions may vary with its states of dryness, temperature, mechanical aggregation, and other circumstances. A light and spongy material will probably be found a more active absorbent of contagion, than the same substance when rendered dense by packing or by manufacturing operations. 3. A low temperature of the porous body will probably cause it to absorb more contagion than an elevated one; the dryness of the solid being supposed equal in both cases. When once impregnated also, an increased temperature will probably act in disengaging fomities, just as odours lurk unperceived in a garment till the wearer enters a warm apartment. It is consistent with this opinion, that clothes, which have been in contact with persons suffering under typhus, sometimes infect those who wash them in hot water. 4. The distance from the source of contagious effluvia, at which porous bodies exert their absorbent power, is undetermined. There is probably a distance at which their elasticity may be so increased by dilution, as to be more than equivalent to the absorbent power of the solid. The more highly the atmosphere surrounding the sick is charged with contagious effluvia, the more abundantly, may it be expected, that those effluvia will be absorbed by solids. 5. The colours of porous bodies have been shown, by the experiments of Dr. Stark, to exert a decided influence over their absorption of odours, the dark colours being most efficient. He has suggested, therefore, by a fair analogy, that colour may modify also the absorption of contagious effluvia.

"XXV. In several well authenticated instances, persons conveying fomites with injurious and even fatal effects to others, have themselves escaped infection. Prisoners discharged in their usual health from Newgate, at the time when that jail was the seat of a contagious fever, have infected the keepers of shops and public-houses in the neighbourhood. The same consequences followed also the liberation of debtors from the jail at Gloucester. In the memorable instance, too, already cited, the criminals who, by the fomites lurking in their clothes, spread so fatal a pestilence through the court of assize, were in their ordinary state of health. Previous ablution of their bodies, and the putting on clean and uninfected clothing, would doubtless have prevented that extensive disaster." (P. 79.)

The consideration of fomites may assist in the solution of a question which has, we think, been rather superfluously agitated, —namely, that of the possibility of infection from dead bodies. Let us suppose the case of a person just dead of a highly contagious disorder. No more of the contagious miasm can be formed, because its formation is doubtless the result of disordered vital actions; but are there no *fomites* about this body which may absorb that which has already been formed? Is the cuticle a less porous body than a piece of cloth; or is the hair of a dead man a worse fomes than a bunch of wool? If not, we do not see how the capacity of the dead body to convey contagion can be disputed, unless on the supposition that the body, when dead, has an active power of neutralizing the miasmata which it evolved when living; an opinion not likely to be maintained.

If numerous instances be adduced where contact with the dead body has failed to communicate disease, this merely shows, what many other facts tend to demonstrate, that contagion is not so universally active as popular apprehension would imply; and the instances where contact with the dead body has been followed by no bad effects might be confronted with a much greater number where similar impunity has attended contact with the living.

II. *Report on Animal Physiology; comprising a Review of the Progress and State of Theory, and of our Information respecting the Blood and the Powers which circulate it.* By WILLIAM CLARK, M.D. F.R.C, F.G.S. F.C. P.S., late Fellow of Trinity College, and Professor of Anatomy in the University of Cambridge.

This paper contains a philosophical survey of the progress of physiological theory, and a condensed view of the improvements which have been effected of late years in some special departments of the science. We shall take the first part alone as the basis of the present notice, since, with respect to the rest, any attempt to epitomize what is itself an abstract would afford little more than a bare enumeration of facts, uninteresting from their want of connexion. In tracing a slight outline of the progress of general physiology, we shall use the licence of modern critics, to make ourselves in a great measure independent of our author; not failing, however, to draw copiously from the excellent materials he has brought together. We may add, that our observations will have less reference to the chronological order of events, than to their influence on the progress of science; and that we shall advert to the discoveries and opinions of individuals only in as far as they bear upon important generalizations.

Physiology has from its commencement been making a kind of double progress. Certain doctrines have arisen from time to time, which, though held in various degrees of estimation at different periods, and variously modified by the advances of knowledge, have never since their origin been entirely lost sight of, but, acquiring increased influence after each season of temporary neglect, have descended to our own times with every prospect of permanence. Other doctrines, again, originating in the partial bias of men's minds, or the defective state of their knowledge at particular periods, have enjoyed only a fitful ascendency; and, though they may have been recalled more than once after their first dismission, they have not returned with augmented strength; they have made no real progress, and are in effect just as far from being verified now as they ever were: but such hypotheses, however erroneous and transitory, have not been without their use, since they have acted as stimulants to industry, and given rise to an immense accumulation of facts, not in themselves the less valuable from having been made subservient to fallacious views, and which, in the gradual progress of science, become involved in just trains of reasoning, and aid in the establishment of true theories. The germ of some of the most important doctrines of physiology is to be found in the writings of the Greeks.

The physiology of Hippocrates was encumbered with the speculations of the Pythagorean school, in conformity with which he taught that the four elements, variously combined, formed the four cardinal humours, and these again the solids and fluids of the animal body; but he inculcated two grand truths, of which one has been very generally received by the best physiologists of every succeeding age; and the other, though not sufficiently recognized even to the present time, will, in all probability, exert an extensive influence on the future progress of the science. The father of medicine perceived clearly that the properties of inert matter would not account for the phænomena of life; he therefore asserted the existence of a power in the living body, which he calls φυσις, *nature.* This he represents as acting instinctively: Ανευρισκει ἡ φυσις αυτη ἑωυτη τας εφοδους ουκ εκ διανοιης. *Nature finds out ways for herself without counsel.* Απαιδευτος ἡ φυσις εουσα και ου μαθουσα τα δεοντα ποιει. *Nature, untaught and without learning, does what is needful. Epidem.*, lib. vi. sect. 5, Ed. Föes.

Hippocrates in effect recognized a vital principle; but he further saw, what it is much to be regretted that so many succeeding physiologists should have lost sight of, that the existence of a living body is one of constant relation to surrounding things; that the vital principle is not self-acting, but a controlling power which regulates the actions excited by the operation of extraneous causes: hence we find throughout his writings the evidences of a constant attention to the effects produced on the animal system by air, moisture, heat, cold, food; in short, by all the external conditions of life which the infant state of natural science at that period offered to his observation.

To Aristotle the science is indebted to an extent which can be appreciated only by those who have made a study of his writings. He cultivated comparative anatomy on a very ample scale, and in a more immediate and comprehensive relation to general physiology than any other enquirer till the time of Hunter. He may thus be said to have instituted the true method of studying the vital functions, since there is scarcely an important fact in physiology which has not been either suggested by comparative anatomy or finally established by an appeal to it.

Aristotle laid the foundation of embryology, by a series of observations on the formation of the chick;* he moreover gave considerable attention to the modifications undergone by each of

* Hist. Animalium, lib. vi. c. 2, 3.

the principal organs of the body, as adapted to the same function in different classes of animals; and, with reference to the lower tribes, noticed the *substitution* of less perfect organs for the performance of similar functions,* so that one might feel inclined to forgive an enthusiastic admirer of the Stagyrite, were he to claim for him the merit of having traced the outline of that great theory of progressive development which has given such an impulse to physiology in the present day.

Aristotle divided the vital powers into the *nutritive* (ϑρεπτικον), *sensitive* (αισθητικον), *appetitive* (ορεκτικον), *locomotive* (κινητικον κατα τοπον), and *cogitative* (διανοητικον).† He referred the first four of these to a kind of soul, common to man, animals, and plants; but regarded the reasoning faculty as appertaining to another kind of soul, capable of a separate and independent existence.‡

This simple and natural classification, when cleared of the logical and metaphysical subtleties in which Aristotle involved it, will be found little at variance with the physiological notions of the present day. The soul common to all organized beings appears to be no other than the vital principle; the nutritive faculty corresponds with organic life; and the combined, sensitive, appetitive, and motive faculties with animal life: in separating the phenomena of the mind altogether from those of the body, and attributing them to a distinct incorporeal being, Aristotle supports an opinion which has always had the ablest, though not perhaps the most numerous, reasoners on its side, and which, notwithstanding the occasional prevalence of different forms of materialism, is, we think, the one most likely to be finally adopted. It is singular that this philosopher, who instituted a very careful and instructive comparison between the mental faculties of brutes and those of man, should have referred the former to the same source as the common functions of life, while he considered the latter as of a nature altogether distinct. It might have been thought that attention to this subject would have convinced so acute a reasoner that the intellectual powers of the inferior animals differ from those of man, not in kind, but in degree.

Galen, perceiving Aristotle's error in associating the phænomena of consciousness and intelligence with those of mere automatic life, attempted a new classification of the vital powers or faculties: these he subdivided into the *natural*, which is centred in the liver, and presides over nutrition, growth, and generation; the *vital*, which has its citadel in the heart, and communicates life and heat, through the arteries, to all parts of the frame; the *animal*, which is seated in the brain, producing the phænomena of the mind, causing sensation and motion by means of the nerves, and presiding over all the other functions: three kinds of spirits, the natural, animal, and vital, formed by a sort of exhalation from the blood,

* The four books De Partibus Animalium, *passim*.

† De Anima, lib. ii. c. 3. ‡ Id. lib. ii. c. 2.

minister to the respective faculties, and convey their influence through the appropriate channels. Besides the three principal faculties, Galen admitted the existence of certain subordinate faculties, which presided over particular organs.

The chief merit of this arrangement is, that it recognizes the distinction between those functions which are essential to the existence of an animal, and those which are only necessary to maintain its conscious relations with external things, and admit of occasional suspension without injury to life.

In his general doctrines, Galen blended the metaphysics of Plato and the logic and natural philosophy of Aristotle, with the physiological principles laid down by Hippocrates; agreeing with the last-mentioned philosopher in deriving the cardinal humours and the various component parts of the animal frame, from the various admixture of the four primary elements. He believed the vegetative or organic faculties to be under the guidance of *nature*, but referred the voluntary to the *soul*. The powerful genius of Galen, aided by a most extensive acquaintance with the philosophy of his age, expanded the science of medicine to an amazing extent, and his vigorous conception brought him to the verge of several important physiological discoveries, which, however, the state of science at that time rendered unattainable. We may, in passing, allude to one point, which has been the subject of some dispute. Galen very decidedly maintained the distinction between the nerves of sensation and those of motion, which had indeed been previously hinted at by Aretæus.* Galen believed that the nerves of sensation were derived from the anterior part of the encephalon, and those of motion from the posterior part, and from the spinal cord: but his idea of the difference between them was purely mechanical: he thought that the former class of nerves were adapted to sensation from their being *soft*, and the latter to motion from their being *hard*. That he had no conception of two distinct kinds of nervous power, resident in different portions of the central mass, is evident from his maintaining that a soft sensatory nerve may become condensed at some part of its course, and thus be converted into a nerve of motion.†

We need not make particular allusion to the Greek writers who succeeded Galen, to those of the Arabian school, or to those who lived soon after the revival of letters in Europe. As far as physiology is concerned, they were all mere copyists: the commanding spirit of Galen overawed the minds of men through many ages of declining science, during which physiologists relinquished all attempts at original observation, and confined themselves to expounding and commenting upon the doctrines of the ancients.

Early in the sixteenth century, the majestic dream of antiquity was broken in upon by the turbulence of the chemists, who,

* De Causis et Signis Diuturnorum Morborum, lib. i. c. 8.

† De Usu Partium, lib. ix, c. 14.

headed by Paracelsus, swore everlasting enmity to the sage of Pergamos, and strove to supplant his specious fallacies by their own less attractive delusions.

In the seventeenth century, a new era dawned upon the science. Harvey's discovery of the circulation of the blood opened a boundless field of enquiry, in which physiologists for some time lost themselves, and wandered in devious and unprofitable paths.

The chemical researches of Hooke and Boyle, and the atomic theory of Descartes, became the basis of those doctrines professed by the chemical and mathematical schools, which now for the first time attracted the general attention of the scientific world.

The chemical sect of physiologists had been founded by Paracelsus in the preceding century, but his peculiar tenets, in which some truth and much ingenuity were blended with a conceit and fanaticism bordering on insanity, sunk into neglect very soon after his death. The school of chemical physicians, however, did not become extinct, and even included in the number of its disciples some individuals eminent for learning and science, among whom Van Helmont deserves to be mentioned.

In the seventeenth century, the improvements in chemistry led to a more plausible application of this science to physiology. Sylvius may be considered as the father of the modern chemical physiology, which derived support from the abilities of Willis, John Mayow, Croone, and Helvetius; but was opposed and confuted by Hoffmann, Boerhaave, and Pitcairn.

Although the views of the chemical physiologists were purely hypothetical, and for the most part chimerical, it is not to be forgotten that they paved the way for the study of animal chemistry: and that the writings of Mayow contain the first hint of the true theory of respiration, and of that solution of the phenomena of animal heat which is most generally received in the present day.

"The mathematical school of physiology," says Dr. Clark, "gained a better reception. Its doctrines, recommended by the prevalence of the atomic theory of Descartes, gave the same direction to physiology and medicine with that in which science was principally advancing under the auspices of the Florentine Academy. The philosophy of Descartes appeared peculiarly applicable to such investigations, since no reason apparently could be assigned which should render that law inapplicable to organic bodies which referred all changes in matter generally to the figure and motion of the ultimate particles of which they were composed. Hence we find the followers of Descartes representing in their works, the mathematical forms of the ultimate particles, of which they supposed the various organs to be composed, as figures for the application of mathematical reasoning. The most distinguished disciple of this school was Borelli. He united to all the anatomical information of the day a depth of mathematical knowledge which enabled him, in appearance, to apply its reasonings and its results to explain the action of the organic machine. Thus, he submitted muscular motion to calculation on the principle of the lever; explained the action of the heart and the motion of the blood upon hydraulic principles; and accounted for the secretions from the various diameters of the vessels. The proximate cause of muscular motion he asserted to be the rush of nervous fluid from the brain upon the muscular fibre. Bellini and Baglivi espoused the same theory, and extended its application by their writings; but, as if internally aware of its insufficiency, and proving that they merely reposed in it as that which was least objectionable, they laboured to separate the theory from the practice of medicine. Thus Baglivi was in practice a follower of Hippocrates and of Sydenham. John Bernouilli was a celebrated disciple of this school. He considered the elementary geometry of the Italians insufficient in its application to the animal body, inasmuch as this represents neither line nor plane either in itself or in the ultimate particles into which it can be resolved. Hence he had recourse to the calculus lately invented by Newton and Leibnitz and the theory of curves. His theory of muscular motion gained great celebrity, as well as his application of the analysis to determine the decrement of the body in consequence of the various transpirations and secretions. Another branch of the mathematical school was founded on the Newtonian theory of attraction, and had for its supporters in this country Keill, the Robinsons, Wintringham, and Meade." (P. 100.)

The mathematical doctrine had, if possible, less foundation in nature than the chemical: it led, however, to much minute dissection and numerous microscopical examinations of the intimate structure of parts; so that, in as far as physiology has received illustration from minute anatomy, it may be considered as indirectly indebted to the mathematical school.

A general recognition of the insufficiency of the hypotheses just alluded to gave origin to a new school of physiology, which has been styled the *dynamic*. Before entering on the consideration of this, however, we may remark that, while the attention of many physiologists had been distracted by futile and short-sighted speculations, there were some who profited by the grand example of Harvey, and devoted themselves to the diligent cultivation of anatomy, with an unprejudiced view to the elucidation of functions. The connexions of the nerves, in particular, were carefully unravelled by Willis and Vieussens; and to the former we owe the first correct view of some of the more remarkable phænomena of sympathy, which had previously been attributed to the continuity of membranes, and other causes inadequate to their production. Willis attributed these phænomena to the agency of the nerves, but erred in supposing that they were referrible to their direct communication. The views of Willis, however, (which were also adopted by Vieussens,) prepared the way for the more accurate opinions of Whytt, who shewed satisfactorily that nervous sympathies take place through the intervention of the great centres of that system; a most important physiological truth, which he has illustrated more fully, and with greater felicity, than any succeeding writer, notwithstanding the metaphysical hypothesis with which he most unnecessarily embarrassed his reasoning.

The dynamic doctrine is usually attributed to Stahl, according to whom the soul is the prime agent in the formation of the body, and the maintenance of all its functions.

A similar opinion had been previously entertained by Van Helmont, who, though of the chemical school, was not the less a vitalist.

The *anima* of Stahl is nearly related to the *Archeus* of Van Helmont; and the latter, again, derived the name and some of the attributes of his vital agent from Paracelsus. It has been thought by some more probable that Stahl borrowed his notion from Hippocrates than from Van Helmont; but the fact of Stahl's being himself enthusiastically addicted to chemistry renders it nearly certain that he must have been familiar with the writings of Van Helmont. Stahl regarded chemistry, mathematics, and anatomy, as equally insufficient to explain the causes of the vital phenomena. According to him, the soul has no particular seat, but is diffused through the whole frame, endowing every organ and texture with its peculiar vital properties, perceiving in the organs of sense, and causing contraction in the muscles, independently of the influence of the brain. The main error of his system consists in the absurdity of an *unconscious intelligence*,—in supposing that the soul can operate and contrive without being aware of it.

In comparing the opinion of Stahl with those of Hippocrates and Hunter, it might at first sight appear that there was little difference among them, except in the name applied to the vital energy: there is, however, this essential difference,—that Stahl considers the *anima* as a distinct being; whereas, Hippocrates and Hunter employ their respective terms, *φυσις* and *vital principle*, merely as the general expression of a number of facts. Stahl's doctrine is based on a false assumption in metaphysics, while Hippocrates and Hunter were content with maintaining that organized bodies present peculiar phænomena which must necessarily have a peculiar cause, without pretending to determine the nature of that cause.

The hypothesis of Stahl received more or less countenance from the writings of Bryan Robinson, Mead, Hartley, Sauvages, Bonnet, and others, who made a partial and restricted use of it; but it found its ablest supporter in Whytt, who fully adopted the tenet that the soul is the cause of all the vital motions. These he divided into *natural*, *involuntary*, and *voluntary*. The natural motions are those which are sustained by the moderate and constant supply of nervous power, uninfluenced by the will or any other stimulus: such are the tension of the sphincters and the general tone of parts;—the involuntary are those excited by stimuli applied to the nerves, in all of which the soul acts automatically and of necessity, but without consciousness;—the voluntary motions are the same as those so designated by other authors. With respect to the first class, it may be observed that Whytt, in common with too many other physiologists, lost sight of the great principle that the state of the living body is one of constant relation to external nature; that numerous extraneous causes are continually operating upon it; and that, consequently, the class of vital motions independent of stimuli is, in all probability, purely imaginary. Again, with respect to the class of involuntary motions, or those in which the soul intervenes without consciousness, if Whytt meant that the soul, even at the moment of its intervention, is actually unconscious of its own operation, the idea is manifestly absurd; since it is impossible to conceive any operation of an intelligent being but as some *mode* of consciousness: but if he meant (as he probably did,) that the action of the soul is so slight and transitory as to leave no trace in the memory even a moment after, what evidence can we have that it has taken place? James Johnstone, with much greater show of reason, referred the involuntary motions to the ganglia of the sympathetic nerve, which he regarded as so many minor nervous centres, sustaining the involuntary functions independently of any immediate connexion with the brain; whence the continuance of these functions when the influence of the brain is suspended by sleep or disease. This opinion, which had been previously hinted at by Winslow and Le Cat, gave rise to the hypothesis which regards the ganglial nerves as a separate system, presiding over the functions of organic life; an hypothesis supported by Bichat and Reil, and by no means subverted, though somewhat weakened, by the experiments of Le Gallois, Wilson Philip, and Magendie.

The chemical, mathematical, and dynamic theories were tempered and combined by Hoffmann and Boerhaave, who were rather judicious critics than original thinkers on physiology. They insisted upon the controlling influence of the nervous system over all the organic functions, whether performed chemically or mechanically. They thus substituted the nervous energy for the anima of Stahl, forgetful of those lower animals in which the existence of nervous matter, though probable, is hypothetical; and of plants, in which it does not exist at all, but both of which are nevertheless endowed with life.

We have now to consider the opinions of Haller, the following judicious review of which we quote from Dr. Clark.

"The great object which that eminent person endeavoured to achieve, was to discover, experimentally, the conditions and the laws which govern those vital phænomena which the assumption neither of mechanical nor of chemical forces had been able to explain, and thus to render physiology as certain as other physical sciences. For this purpose he excluded those metaphysical subtleties by which his predecessors had so frequently veiled ignorance; excluded also mathematical and chemical science in all cases in which it was impossible to ascertain the elements upon which their application could be founded. He was willing, as he himself says, to confess himself ignorant of the manner in which the soul and body are united, and was content to proceed no further than those discoverable laws which the Creator has himself prescribed, without inventing others unwarranted by experience. On this principle he instituted innumerable experiments to discover and illustrate the properties of the vital powers. He proved the existence of a property in muscle, to which he restricted the term *irritability*, which is only called into action by means of stimuli, which affects a much greater vivacity of motion than mere elasticity (a property of dead matter), the motions also consisting in alternate oscillations, with contraction, swelling, and wrinkling of the fibre, followed by extension, relaxation, and elongation of the same. He further attributed to the muscles a *nervous power*, distributed to them from the brain by means of the nerves, as a necessary condition of their irritability, but which entirely differs from it. He concluded from his experiments, as detailed in his earlier works, that the following parts are destitute of irritability and nervous power: periosteum, peritoneum, pleura, ligament, tendon, articular capsules, the cornea, parenchyma of the viscera. In these tissues he admitted a force analogous to elasticity, inherent in their organic texture, which solicits them to contract slowly when divided, when exposed to cold, &c., and which only abandons them when entirely disorganized. He proved that sensibility is inherent in the nerves, but that they are destitute of irritability. He denied that irritability could be imparted to the muscles by the nerves, because, seeing that a nerve, on being stimulated, may excite motion in the muscle to which it passes, but offers not the slightest motion itself, it is impossible to suppose they should be the source of that to others which they never possessed themselves; and, more particularly, because he perceived that the excitement of muscles through nerves is a phænomenon not true of all, but only of certain muscles.

" He proved, universally, that irritability resides in all parts that have muscular fibre; that this power differs in intensity and permanence in various parts; that these qualities are most observable in the heart, more in the left ventricle than the right; that next in order come the intestines, the diaphragm, the voluntary muscles. From reiterated experiments he concluded that the heart and other involuntary muscles are not excited to contract by stimulating the nerves with which they are supplied, but that they require specific stimuli: thus, that the blood is to the heart what the will is to the voluntary muscles.

" In this way Haller restricted the vital powers to two,—sensibility and irritability; the one exhibited in the brain and nerves, the other in muscular fibre. His doctrine was vehemently opposed by Whytt, De Haen, Verschuir; and strenuously defended by himself, by Bonnet, and by Fontana. It was seen that many parts in the animal body to which neither irritability nor sensibility, in Haller's sense, could be extended, were not the less alive. Thus during the numerous controversies which arose, errors on each side were detected; materials for more extended views were accumulated; experiments were infinitely multiplied and eagerly criticised; the excitability of various tissues, to which Haller had denied that quality, because he had not called it into action by an appropriate stimulus, was established on the one hand, and on the other the mistake of confounding nervous influence with sensibility was made apparent. Thus the more probable it became that irritability and innervation are separate powers, so did it follow the more necessarily that every different part should have its own excitability and its own degree of nervous power, and hence its own peculiar mode of life,—an opinion announced by Bordeu, Barthez, Blumenbach. Independently of these expressions of vital energy in the various tissues, these physiologists admitted a fundamental power, which they termed *vitality*, or *vis vitæ*, of which the different degrees of excitability and sensibility were considered merely as modes, according to the organs in which the vital energy operated. But the analogies thus assumed between the phænomena were not established by any proof; the modifications of the original power were not accounted for; and this theory, apparently philosophic, has no firm foundation when its partisans would represent vitality, or oxygen, or galvanism, as a proximate cause of all the phænomena, residing in living matter as gravity does in dead." (P. 103.)

Haller, then, established the general proposition that peculiar modifications of vital power are connected with, or inherent in, particular tissues; a principle of the greatest utility, as tending to obviate those attempts at personifying the vital energy which have always distracted the attention of physiologists from the actual phenomena of life.

The study of General Anatomy, commenced by Andrew Bonn, and raised to importance by Bichat, extended the materials for the application of Haller's views. The researches of Bichat, however, have not hitherto exerted the influence on physiology which might have been expected from so fine a generalization. This has probably arisen from two circumstances: first, that the doctrine of the tissues has been incautiously applied, in some instances by Bichat himself, and in others by his successors; mere continuity of surface having been too hastily received as evidence of similar vital endowments, and a number of mixed tissues thus artificially combined into one, supposed to be simple and homogeneous: the mucous membranes may be adduced as an example. Secondly, that the anatomical part of the enquiry has not yet been pushed to the limits which it is evidently capable of attaining:—to this point we shall presently recur. A few years previous to the appearance of Bichat's work on general anatomy, John Hunter had promulgated his grand doctrine of the life of the blood,* which regards this fluid as endowed with vitality, as well as the solid textures: perhaps, indeed, as the original source from which every

* In attributing this doctrine to Hunter, we by no means intend to assert that he was the first who taught that the blood was endowed with life, though the limited extent of his own reading induced him to believe so. This opinion was strongly, nay enthusiastically, maintained by Harvey, who has, however, fallen into so many contradictions and absurdities with regard to it, as to divest it of all consistency and applicability: thus, at one time he considers the blood as the instrument of the soul, and at another as the soul itself; sometimes the soul is merely an act of the blood, at others it is the *calidum innatum*; and at others, again, the soul is held to be pre-existent, and to generate both the blood and the calidum innatum. Hunter's doctrine, on the contrary, is simple and intelligible: he merely contends that the blood is endowed with vitality as well as the solid parts, and supports his position by experiments and observations, which, if maturely considered, leave little room for doubt upon the subject.

modification of vital power is derived. For some time the experiments and reasoning of Hunter appear not to have induced very general conviction: further research and reflection have, however, dispelled the doubts of physiologists, and the vitality of the blood is now almost universally recognized: many even suppose that its red particles are capable of spontaneous motion, an opinion which is probably erroneous.

Physiology had now advanced nearly as far as the paths of enquiry hitherto pursued could be expected to lead it; but, as human knowledge is destined to be progressive, new sources of information quickly suggest themselves when the old ones are exhausted. As yet the vital functions had been considered in their perfect state; the materials for a different method of investigation had, however, long been accumulating, and it now seems to have occurred to physiologists that the facts of embryology might throw light on these functions, by pointing out their simplest, that is, their most essential conditions, and tracing the gradations by which they arrive at greater complexity. Hence arose the theory of *development*, which, originally hinted at by Harvey, if not by Aristotle, has grown, under the hands of Hunter, Wolff, Prevost and Dumas, Meckel, Tiedemann, Serres, St. Hilaire, and Von Baer, into a doctrine of the highest interest and importance.

Dr. Clark has given an admirable view of this doctrine, and of the reasoning by which Professor Tiedemann has connected the development of the living organism with the *assimilative process*.

"The earliest examinations that can be made of plants or of animals present them as consisting of a minute globule of fluid, or a minute disc of slightly albuminous matter, i. e. under aspects not distinguishable in different future genera or species, as to properties or forms of their matter, by any tests which we possess. In the near neighbourhood of the disc is placed, in animals, a quantity of nutritive substance, by means of which it is destined to work. The effects, when produced, are definite for each species: but none occur except under certain conditions. These conditions are, a due degree of moisture, of air, and of warmth. When they are supplied, the disc is capable of being affected by the matter in its neighbourhood. It is *excited*, and it *reacts*. The consequence of the reaction is a gradual expansion of the disc to surround the nutrient matter; a separation of it into different superposed portions, which come into view; and a gradual appropriation of the nutrient matter. Upon the external portion of the disc, the first trace of the nervous system is observed; upon the internal portion, that of the intestinal canal; intermediate between them, that of the vascular system. Though at first simple, these objects have still a certain magnitude, and the later more complex formations are seen to arise from them as if by vegetation. 'The first trace of the nervous system is not merely that of the spinal cord or of the ganglionic string, but is the potential whole of that system, of the brain and all its appendages. The first trace of the abdominal canal is not merely the rudiment of that canal, but of the whole glandular apparatus also, which may be seen gradually to spring from it.' And thus is the observed process of development altogether contradictory of the theory of Haller and of Bonnet, which represents each organ as absolutely existing in the germ, though in a miniature form. That the power which effects these changes, and thus controls the disposition of organic molecules, resides in the disc, is ascertained from the facts, that ova belonging to species the most different are all developed, according to their kinds, under similar external conditions, and that ova of the same species are true to their kinds under conditions which are not absolutely the same for any two individuals. If we call this power *vitality* with modern writers, or the *anima* with Stahl, these words can teach us nothing physiologically, unless we ascertain the law by which it operates: however we may see that the final cause of its operation is plainly in every case the production of those numerous bodies, definite with respect to families, genera, and species, which it developes for its own manifestations in each. Our eyes inform us that these bodies arise by means of the assimilative process, and that the original power exhibits its faculties by means of the organs which it has produced through this process. Our idea then of the vital power is this,—that it is connected with the matter of the germ in the act of its formation, and resides in it as the potential whole, or sufficient cause, of the entire future organism; that in consequence of the excitability of the organic matter of the germ, imparted to it in the same act of its formation, the expansion of the germ into portions or members occurs by the visible process of assimilation or nutrition, each portion thus acquiring its own excitability and its own reactive energy, which are but partial manifestations of the original power; and that in proportion as each part is developed, new internal conditions are introduced, in consequence of the new formation, which affect all that previously existed, by modifying the assimilative process in all. The phases of this process are strictly defined for each species, and the subsidiary means necessary for the purposed effect—as in the various forms of the respiratory organ in the fœtal state of the same individual to moderate the condition of external air, are amongst the most beautiful instances of provision for a definite end.

"This formative act, this process of assimilation or nutrition, which is thus performed by animals and plants, and has a relation not only to the present, but the future also, appears to be the determination of a power acting according to Reason; and hence it must have been that Stahl referred it to the rational soul. But, seeing that reason cannot exist without consciousness, a faculty which manifests itself only by means of the brain, a late product of this very power by the act of assimilation,—seeing also that the effect may be modified, within limits, (as in cases of monstrosity,) when the conditions are altered, we rather conclude with Harvey that it proceeds from a power acting according to fixed laws. "Vegetativæ operationes potius videntur arte, electione et providentiâ institui, quam animæ rationalis mentisve actiones; idque etiam in homine perfectissimo.'

"A peculiar matter is necessary for the manifestation of vital phænomena: this matter is called *organic*. It is not the cause of life, but rather is its act, a production by means of the assimilative process, for the exemplification of the allotted faculties. The faculty is imperfectly manifested if the organ be imperfectly formed: the organ and its energy both vary with variations in the nutritive process.

"Hence those subordinate expressions of vital force, called *nervous power, force of secretion*, &c., cannot be considered as distinct and independent powers. They are produced, or evidenced, with their organs, by the force of assimilation, and are maintained by the same. They depend upon it for their manifestation and their due support.

"Vital power imparted to organic matter (which is itself the product of the living power of the parents), and exemplifying its faculties by means of the organs which it has developed through the force of nutrition, seems to be the last step to which observation and induction has hitherto led us. The induction is verified by observation. If the assimilative process be altered in any organ, the expression of excitability and of vital reaction peculiar to that organ is altered in the same degree.

"There are then in living bodies as many species of excitability and as many modes of reaction as there are tissues. Every one of these has its own mode of both, which is called into action by its own appropriate stimuli. 'Whatever these stimuli may be,—whether external, as air, light, warmth, food; or internal stimuli, the blood, nervous influence, the secreted humours,—each organ reacts in its own peculiar manner: a manner which supposes a peculiar organic power imparted to it in the act of its formation by the process of nutrition, and sustained by the same.' 'The stimulus may be that of a chemical, or mechanical, or organic substance; the reaction, however, is always vital, and indicates the existence of an organic force, of which it is the effect. The physical properties of the one are in some sort in a constant conflict with the vital properties of the other, and living bodies only preserve their character of life so long as they are able to resist the physical impression. When it is said that organic movements are occasioned by incitations, we do not admit that they are the immediate effects of the mechanical or chemical impressions, but assert that they are the effects of powers which the external impression, be it mechanical or be it chemical, has thus solicited to act.' (P. 106.)

It may be added, that the younger St. Hilaire has extended the doctrine of development by some ingenious ideas as to the expansion of some particular organs and systems, which follow a progress of their own, in some degree independent of the entire organism, but having certain determinate relations to its growth. The most remarkable example is to be found in the reproductive system, from the development of which arise the phenomena of puberty.

The study of comparative anatomy, of which Aristotle was the founder, and which John Hunter and Cuvier have revived on so magnificent a scale, illustrates, throughout living nature, a series of developments analogous, though not parallel, to those of the individual organism. This analogy has been strained by some imaginative physiologists into nothing less than the hypothesis that the fœtus of the higher animals passes in succession through all those states of organization in which the inferior tribes permanently exist; the human fœtus thus presenting a kind of historical summary of the progress of life and organization, from the beginning of all things; an hypothesis in which it is too evident that the generalization has preceded the full investigation of the facts, since these, though tortured into a partial conformity in some instances, offer a decided and conclusive opposition in others.

Comparative anatomy, when aided by geology, becomes retrospective, and exhibits a vast series of living things adapted to the condition of the earth's surface at the several periods of their existence, and gradually advancing from simpler to more complex forms of organization: hence some physiologists have surmised that all the varieties of organic structure may be mere modifications of a simple and primary form; an hypothesis commending itself to the imagination by the grandeur of a conception which regards the organic kingdom as one colossal system of development, wherein the earliest form of life is the embryo, and the human frame its full evolution. On this subject Dr. Clark has the following just observation:

"The undoubted fact that existing species have been perpetuated unchanged for several thousands of years, would have rendered such an opinion in the highest degree improbable, but for the observations relative to the apparently spontaneous production of animals and of plants from organic matter in solution—the apparent changes of species from simpler to more complex under favourable external circumstances—and the interchange of animal and vegetable form. If the facts were really thus, then might the objection to the hypothesis of metamorphosis founded on the permanence of existing forms be encountered. It might be averred that, notwithstanding our ignorance of the means, the necessary conditions for such successive changes may have been supplied in the earlier periods of the world, at epochs so far removed, that the few thousands of years which have passed away since the appearance of man upon the globe bear no proportion to their immense distance, and only show that the rate according to which the conditions of change are produced is a very slow one." (P. 110.)

The belief in equivocal generation, universally adopted by the ancients, was thrown into discredit by the experiments of Redi and Valisnieri; it derived new support from those of Tuberville Needham, Müller, and Wrisberg; it was controverted by Spallanzani, and since his time has been the subject of divided opinions. Priestley observed that the green matter produced in organic infusions by exposure to light was at first a mass of animalcules; that it was then resolved into green globules, which concreted into confervæ; and lastly, after the solution of these, that it was again converted into infusory animals and vegetables. The organic particles thus appeared common to animals and vegetables, entering into the formation of either according to circumstances, and retaining their own independent vitality when the organisms they had formed were dissolved. These observations have been confirmed by Ingenhouz and Treviranus; but the recent researches of Ehrenberg are opposed both to the spontaneous origin of infusory animals and the convertibility of animal and vegetable life. The two points, therefore, on which the hypothesis of development from one primary form mainly depends, are still disputed: the hypothesis, however, though on the whole improbable, deserves to be kept in view.

Comparative anatomy, like other great sources of physiological truth, must be expected, in the earlier periods of its application, to give rise to some chimerical views; but, when placed in its true relations to the science of life, by the exercise of a cautious philosophy, is likely not only to afford the most ample materials for the investigation of the vital phenomena, but the most effectual safeguard against partial views and hasty conclusions. It must henceforth be considered as the grand basis of physiology: human anatomy has long been acknowledged as such; comparative anatomy is merely its extension, on which a larger and more durable superstructure is to be reared.

The doctrine of peculiar vital powers resident in particular textures has been applied to the nervous system only of late years: indeed, the apparent homogeneity of the nervous substance afforded little ground for suspecting a difference of intimate organization and of vital power in the several parts which compose this system.

Bichat recognized two nervous tissues, appropriated respectively to animal and organic life; but it is to Sir C. Bell that physiology is indebted for the idea that each variety of nervous power has its origin in some defined portion of the central mass; and his own researches, with others to which they have given rise, have established those important facts which are too familiar to require explanation here. The separate functions of the anterior and posterior columns of the spinal cord are now universally admitted, and Sir C. Bell's views of the respiratory system of nerves, though less evidently demonstrated, are pretty generally received by physiologists. An attempt has been made by Dr. W. Philip, (though, as we think, very unsuccessfully,) to prove that respiration is a purely voluntary function. This eminent physiologist seems here to have fallen into the same error as Whytt; he has made his hypothesis hinge on a point in metaphysics that can never be settled.

The relation of the nervous to the vascular system has received illustration from the experiments of Le Gallois, W. Philip, Flourens, Marshall Hall, and others. The most important conclusions to be derived from these researches are thus luminously stated by Dr. Clark.

"Haller, from observing that the heart continues to beat for a considerable time even when removed from the body; and that its contractions, in the body, may be affected by the direct application of mechanical and chemical stimuli to its fibres, whilst he could not influence them by irritation of the cardiac nerves, concluded that its power of contraction is inherent, and totally independent of the nervous system. His theory was afterwards fortified by the dissections of Sœmmerring and Behrends, which appeared to show that the cardiac nerves are distributed to the vessels of the heart alone. And even after Fowler, Humboldt, and others had stated that the heart may be stimulated by galvanizing its nerves, and Scarpa had demonstrated that these are distributed to its substance as in other muscles, Haller's theory, though vehemently opposed at first, came to be very generally received. It, however, met a formidable opponent in Le Gallois, who published, in 1812, an essay, containing results of numerous experiments, from which it appeared that the heart's power is altogether derived from the spinal cord. He found, that if a rabbit be decapitated, the heart's action is continued, artificial respiration being performed; that if a portion of the cord be destroyed, as in the lumbar region, the heart is unable to support the circulation, in a rabbit twenty days old, longer than four minutes, whilst it is continued in one two days old; and that the destruction of the cervical and dorsal portions of the cord are still more suddenly fatal to the heart's action. He observed, on destroying successive portions of the cord, that even when the circulation is suddenly arrested, life ceases, on the instant, only in those parts which derive their nerves from that portion of the cord which has been destroyed, continuing for a time in the rest of the body; that this time is greater the nearer the animal is to the epoch of its birth, and is determinate for each species. He concluded that those parts which die last, on partial mutilation of the cord, die because the power of the heart has been so much weakened that the circulation through the entire arteries cannot be maintained. He hence inferred, that if the work to be performed by the heart were diminished in proportion as its power was lost, the circulation might be supported. He found, accordingly, that if the aorta was tied opposite to the part of the cord to be destroyed, the circulation was continued through the remaining portion of the trunk in connexion with the heart. His general conclusions were, that the heart has no intrinsic power, but that it derives its power from every part of the spinal cord; that each part of the body is animated by that part of the cord from which its nerves arise; that the sympathetic system of nerves has its origin in the spinal cord, and not in the ganglia, its office being to bring the parts to which it is distributed within the influence of the whole nervous power of the cord; that the motions of the heart which are visible after excision from the body, are similar to those which may be excited in other muscles after they have been for some time dead, and are merely cadaveric phænomena." (P. 137.)

These conclusions are strengthened by a reference to comparative anatomy, which teaches us that the vascular and nervous system advance together towards perfection, arguing an intimate relation between them; but that the former exists earlier in the scale of organization than the latter, thus demonstrating the independence of its vital endowments.

The hasty glance that has been thrown over the progress of physiology is sufficient to afford some useful inferences as to its leading principles, and the direction in which further research may be most profitably pursued.

1. The manifest insufficiency of chemistry and mechanical philosophy to account for the phenomena of life, has led to a general recognition of a power in living animals, acting according to laws different from those of inert matter. Whenever physiologists have lost sight of this truth, they have been led into inane and even ridiculous speculations. On the other hand, all attempts to define the nature of this power,—whether it reside in one agent or in many,—whether it be a property of some particular kind of matter, or something superadded to common matter,—have led to hypotheses perhaps only less repugnant to common sense from coming less under its cognizance, and certainly not approximating at all more closely to the truth.

All that we know of vitality is, that it exists and manifests itself by phenomena differing in each variety of organic texture. Our actual acquaintance with the extent and connexions of the animal tissues is extremely limited, and physiology here calls for the aid of minute anatomy, which, if assiduously cultivated, will probably give increased applicability to Haller's doctrine.

The tissues have hitherto been considered chiefly in their expansion over large surfaces: it seems to have been forgotten that the parenchyma of the viscera most essential to life is entirely composed of an involution of these tissues, and, till the structure of every organ has been developed by the careful unravelling of the tissues that enter into its formation, physiologists must not expect that general anatomy can be brought into any very instructive relation to their science. The enquiry above proposed has been lately commenced by Mr. Kiernan, whose investigation of the intimate structure of the liver has been conducted on the strict principles of general anatomy: the perusal of his admirable memoir has suggested the preceding observations, and we hope to see the anatomy of many other organs investigated by him in the same masterly manner.

2. The vital principle is not self-acting, but is excited into activity by an immense number of extraneous causes, of which the operation on the organism is so controlled and modified by the presiding power, as to produce those phenomena the aggregate of which is called life.

We have remarked that this important consideration appears to have been more present to the mind of Hippocrates than of many physiologists of modern times; in fact, it seems to have been nearly lost sight of till brought into notice by the theory of development, by Tiedemann's ideas on assimilation, and by the highly philosophical attempts of De Blainville to establish valid distinctions between organized and inorganic bodies: it could never have been disputed, (for its truth is at once obvious,) but it was not taken into account, and its absence from the speculations of physiologists may have been one cause of the disposition to personify the vital principle, to endow it with reason, and to invent divers idle hypotheses of life, instead of investigating its declared laws, and the conditions under which it is found to exist.

The relation between life and its external conditions, once placed in a prominent point of view, cannot fail to engage a large share of the future attention of physiologists. The advanced state of the natural sciences affords a considerable store of materials for the prosecution of this subject, which has already been partially illustrated by writers on the natural history of man and animals, and on medical topography and statistics: such researches, though hitherto conducted without reference to their most important tendency, have pointed out many striking examples of the influence of external causes in diversifying the phenomena of life, and in modifying, nay almost metamorphosing, the forms of organization.

The living body, thus inseparably connected with external nature, is not stationary, but passes through successive stages of growth, maturity, and decline. In these stages, its relations to surrounding things undergo a gradual change: the fœtus, the growing child, the youth, the adult, and the aged man, have each their peculiar conditions of life; some of which it would not be difficult to enumerate, and all of which, as far as they can be observed, should be subjected to a much more careful consideration than they have yet obtained.

3. Not only is the entire organism kept in a state of constant relation to external nature, but the several systems which compose it maintain a relation among themselves, which varies at different periods of existence: thus, the vascular and nervous systems preserve a balance essential to the continuance of life, and the reproductive organs acquire, at a particular epoch, a preponderance of action which modifies to a greater or less extent the whole range of its phenomena.

The influence of particular systems and organs on the vital manifestations of the entire organism have hitherto been chiefly investigated by the mutilation of animals; a mode of enquiry which can never lead to satisfactory results. Such experiments rather demonstrate how the phenomena of life are enfeebled or annihilated by the abrogation of a function, than how they are sustained by its exercise. Death, not life, becomes the subject of observation.

The anatomical comparison of organized beings affords a more promising method of enquiry: it presents us with subjects deprived of certain organs and functions, without detriment to life; it disjoins systems, and re-unites them in different degrees of perfection, thus supplying us with data for synthetical as well as analytical observations.

